# Physapruin A Induces Reactive Oxygen Species to Trigger Cytoprotective Autophagy of Breast Cancer Cells

**DOI:** 10.3390/antiox11071352

**Published:** 2022-07-11

**Authors:** Tzu-Jung Yu, Jun-Ping Shiau, Jen-Yang Tang, Chia-Hung Yen, Ming-Feng Hou, Yuan-Bin Cheng, Chih-Wen Shu, Hsueh-Wei Chang

**Affiliations:** 1 Graduate Institute of Natural Products, Kaohsiung Medical University, Kaohsiung 80708, Taiwan; u109831002@kmu.edu.tw (T.-J.Y.); chyen@kmu.edu.tw (C.-H.Y.); 2 Division of Breast Oncology and Surgery, Department of Surgery, Kaohsiung Medical University Hospital, Kaohsiung 80708, Taiwan; 1060526@kmuh.org.tw (J.-P.S.); mifeho@kmu.edu.tw (M.-F.H.); 3 Department of Surgery, Kaohsiung Municipal Siaogang Hospital, Kaohsiung 81267, Taiwan; 4 School of Post-Baccalaureate Medicine, Kaohsiung Medical University, Kaohsiung 80708, Taiwan; reyata@kmu.edu.tw; 5 Department of Radiation Oncology, Kaohsiung Medical University Hospital, Kaohsiung 80708, Taiwan; 6 Department of Biomedical Science and Environmental Biology, College of Life Science, Kaohsiung Medical University, Kaohsiung 80708, Taiwan; 7 Department of Marine Biotechnology and Resources, National Sun Yat-sen University, Kaohsiung 80424, Taiwan; jmb@mail.nsysu.edu.tw; 8 Institute of BioPharmaceutical Sciences, National Sun Yat-sen University, Kaohsiung 80424, Taiwan; 9 Center for Cancer Research, Kaohsiung Medical University, Kaohsiung 80708, Taiwan

**Keywords:** withanolides, oxidative stress, autophagy, breast cancer

## Abstract

*Physalis peruviana*-derived physapruin A (PHA) is a potent compound that selectively generates reactive oxygen species (ROS) and induces cancer cell death. Autophagy, a cellular self-clearance pathway, can be induced by ROS and plays a dual role in cancer cell death. However, the role of autophagy in PHA-treated cancer cells is not understood. Our study initially showed that autophagy inhibitors such as bafilomycin A1 enhanced the cytotoxic effects of PHA in breast cancer cell lines, including MCF7 and MDA-MB-231. PHA treatment decreased the p62 protein level and increased LC3-II flux. PHA increased the fluorescence intensity of DAPGreen and DALGreen, which are used to reflect the formation of autophagosome/autolysosome and autolysosome, respectively. ROS scavenger *N*-acetylcysteine (NAC) decreased PHA-elevated autophagy activity, implying that PHA-induced ROS may be required for autophagy induction in breast cancer cells. Moreover, the autophagy inhibitor increased ROS levels and enhanced PHA-elevated ROS levels, while NAC scavenges the produced ROS resulting from PHA and autophagy inhibitor. In addition, the autophagy inhibitor elevated the PHA-induced proportion of annexin V/7-aminoactinmycin D and cleavage of caspase-3/8/9 and poly (ADP-ribose) polymerase. In contrast, NAC and apoptosis inhibitor Z-VAD-FMK blocked the proportion of annexin V/7-aminoactinmycin D and the activation of caspases. Taken together, PHA induced ROS to promote autophagy, which might play an antioxidant and anti-apoptotic role in breast cancer cells.

## 1. Introduction

Breast cancer is the primary cancer cause of death in women based on 2020 global cancer statistics [1]. Female breast cancer takes 11.7% of all cancer occurrences [1]. Several molecular markers were reported, such as estrogen receptor (ER), progesterone receptor (PR), and human epidermal growth factor receptor 2 (HER2) [2]. Most breast cancer exhibits positive expression for some of them. Some aggressive types lack these markers and take 10–15% of breast cancer, called triple-negative breast cancer (TNBC) [3]. Although several chemotherapeutic drugs and targeted therapies have been used to treat breast cancer, the mortality rate has increased worldwide in the past 25 years [4]. Thus, it is essential to develop more anticancer agents against both non-TNBC and TNBC.

*Physalis peruviana* L. is an edible Solanaceae plant [5] and functions as a traditional medicine in Asia and South America [6]. *P. peruviana* is rich in withanolides [7] and shows anticancer effects [8,9,10,11]. Physapruin A (PHA), a *P. peruviana*-derived withanolide, can inhibit the proliferation of breast cancer by triggering oxidative stress, DNA damage, and apoptosis [12]. However, other non-apoptosis signaling pathways were not investigated.

Oxidative stress can regulate several cellular functions, such as apoptosis, DNA damage, endoplasmic reticulum stress, and autophagy [13]. Reactive oxygen species (ROS) can trigger autophagy in several cancers, such as leukemia [14]. Autophagy (self-eating) is an intracellular metabolism process where damaged organelles are recycled for energy production under nutrient deprivation or cell stress [15]. ROS may modulate autophagy, which controls cell survival and death [16]. The interaction between ROS and autophagy in tumor initiation and cancer therapy has been reported [17]. Modulating oxidative stress has been reported to regulate autophagy in cancer therapy [18,19]. However, the impact of PHA on modulating autophagy in breast cancer cells remains unclear.

Some cancer studies [20,21] showed an interactive relationship between apoptosis and autophagy. Some drugs modulate both apoptosis and autophagy. For example, a synthetic oleanolic acid derivative, SZC015, induced apoptosis and autophagy in breast cancer cells [22]. Ophiobolin A, a sesterterpenoid fungal phytotoxin, induced autophagy and apoptosis in melanoma cells [23]. Corynoxine B, a natural alkaloid, suppresses apoptosis and enhances autophagy to improve cell proliferation of pheochromocytoma cells [24]. Isoaaptamine, a marine sponge-derived compound, triggers apoptosis and autophagy of breast cancer cells mediated by oxidative stress [25]. However, the relationship between autophagy and apoptosis in PHA-treated breast cancer cells remains unclear.

The present study investigated the impact of PHA-generated ROS on autophagy activation and its modulating effects on apoptosis in breast cancer cells. The impact of oxidative stress in regulating autophagy was also assessed. 

## 2. Materials and Methods

### 2.1. Cell Cultures and Reagents

In the American Tissue Culture Collection (ATCC, Manassas, VA, USA), human breast cancer cell lines MCF7 and MDA-MB-231 were used. They were cultured with Dulbecco’s Modified Eagle Medium (DMEM)/F12 (3:2 mixture) mixed with 10% bovine serum (Gibco, Grand Island, NY, USA) and supplemented with P/S antibiotics and glutamine. The cells were kept in a humidified incubator with 5% CO_2_ at 37 °C.

PHA was purchased from BioBioPha Co. (Yunnan, China). It was further confirmed by a Varian 400 MHz NMR spectrometer. The ^1^H NMR spectrum (Figure S1) matched the data for PHA in our previous work [12] and showed high purity (no impurity peaks). Inhibitors of autophagy, free radicals, and apoptosis such as bafilomycin A1 (Baf A1), *N*-acetylcysteine (NAC) [26,27,28,29] (Sigma-Aldrich, St. Louis, MO, USA), and Z-VAD-FMK (ZVAD) (Selleckchem.com; Houston, TX, USA) were chosen as pretreatments before drug treatments.

### 2.2. Cell Viability

Cell survival was examined by the ATPlite luminescence product (PerkinElmer Life Sciences, Boston, MA, USA) [30]. According to the user manual, cell lysate was incubated with the substrate in darkness for 5 min and read by a luminometer (Berthold Technologies GmbH & Co., Bad Wildbad, Germany).

### 2.3. Autophagy Activation and Influx

Autophagy signaling protein expressions were determined by Western blotting [31]. Autophagy antibodies included p62 and LC3A/B (Cell signaling, Danvers, MA, USA). β-actin (Sigma-Aldrich; St. Louis, MO, USA) was used to detect the loading control. The net change of LC3B-II [32] levels between treatment, with and without the autophagy inhibitor, (Baf A1) was counted as autophagy flux, i.e., ((PHA and Baf A1 − PHA))/(Baf A1 − control).

Moreover, autophagy was also detected by flow cytometry. DAPGreen was designed to detect autophagosomes and autolysosomes, whereas DALGreen was intended to detect only autolysosomes. Cells were incubated with DAPGreen dye [33] (0.1 μM) or DALGreen dye [34] (0.5 μM) (Dojindo, Kumamoto, Japan) at 37 °C for 30 min. After washing with PBS twice, cells were treated with PHA or NAC/PHA at 37 °C for 6 h. Finally, they were detected by using a Guava easyCyte flow cytometer.

### 2.4. ROS Measurement

Cells were processed with a nonfluorescent 2′,7′-dichlorodihydrofluorescein diacetate (H_2_DCFDA) staining (Sigma-Aldrich) at 10 μM (37 °C, 30 min) in darkness [30]. The ROS-activated dye became fluorophore and was detected by the Guava easyCyte flow cytometer. Its intensity was calculated by using FlowJo software.

### 2.5. Quantitative RT-PCR (qRT-PCR)

Total RNA was converted to cDNA as described previously [35]. qRT-PCR was performed by the CFX Connect real-time machine (Bio-Rad) (Bio-Rad Laboratories, Hercules, CA, USA), which was ran by using a touch-down program [35]. The antioxidant-associated genes were tested as follows [36]: Superoxide dismutase 1 (*SOD1*), nuclear factor erythroid 2-like 2 (*NFE2L2*), thioredoxin (*TXN*), and glutathione-disulfide reductase (*GSR*) (Table 1). mRNA expression was estimated by the 2^−ΔΔCt^ method [37] compared to the *GAPDH* gene [38].

### 2.6. Apoptosis Detection

Apoptosis was determined by annexin V/7AAD, Caspase-Glo^®^ 3/7, and Western blotting as follows. Annexin V-FITC (1:1000)/7AAD (1 μg/mL) kit (Strong Biotech Corporation, Taipei, Taiwan) was added to cell suspensions at 37 °C for 30 min and was applied to a flow cytometry analysis [30].

The apoptosis executing enzyme caspase 3/7 (Cas 3/7) was activated by apoptosis. The activity of Cas 3/7 was determined by Caspase-Glo^®^ 3/7 kit (Promega; Madison, WI, USA). The Cas 3/7 tetrapeptide substrate (DEVD) could potentially react with active Cas 3/7. After cutting by active Cas 3/7, DEVD became a luminogen, and a microplate luminometer measured its intensity.

Apoptosis signaling protein expressions were detected by Western blotting. Apoptosis antibodies included cleaved poly (ADP-ribose), polymerase (c-PARP), and caspases 3, 8, and 9 (c-Cas 3, 8, and 9) (Cell signaling). Β-actin (Sigma-Aldrich) was used to detect the loading control [39]. The remaining information was mentioned previously [30].

### 2.7. Statistical Analysis

The significance of multi-comparisons was analyzed by a one-way ANOVA and Tukey HSD test (JMP software; SAS Institute Inc., Cary, NC, USA). The statistical software provided the lower case letters for each treatment, which judged the significance between them. Different treatments showed non-overlapping letters, which revealed significant differences.

## 3. Results

### 3.1. Downregulating Autophagy Enhances Cytotoxic Effects of PHA in Breast Cancer Cells

**Figure 1:**
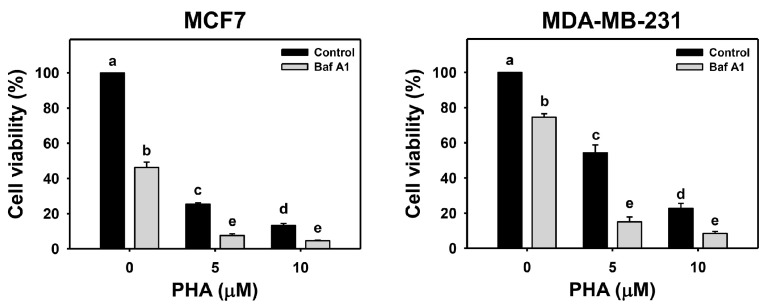
Autophagy downregulation reduces the cell viability of PHA-treated breast cancer cells. Cell viability was assessed by ATP level after 24 h of drug treatment. Breast cancer cells (MCF7 and MDA-MB-231) were co-treated with PHA (0 (control), 5, and 10 μM) and Baf A1 (0 (control) and 100 nM) for 24 h, namely Baf A1/PHA. Data were expressed as means ± SDs (*n* = 3). Data with non-overlapping lower-case letters differ significantly (*p* < 0.05 to 0.0001).

The viability of breast cancer (MCF7 and MDA-MB-231) cells was dose-responsively decreased by PHA treatment in 24 h according to cellular ATP levels. Autophagy could be beneficial and detrimental for cells in response to stress. An autophagy inhibitor was initially used to inspect the involvement of autophagy in PHA-induced cytotoxicity. Cotreatment with an autophagy inhibitor (Baf A1) and PHA displayed lower viability than PHA treatment. Accordingly, autophagy inhibition enhanced the PHA-induced cytotoxicity of breast cancer cells (Figure 1), suggesting that autophagy might play a protective pathway in breast cancer cells when exposed to PHA.

### 3.2. PHA Induces Autophagy and Autophagic Flux

**Figure 2:**
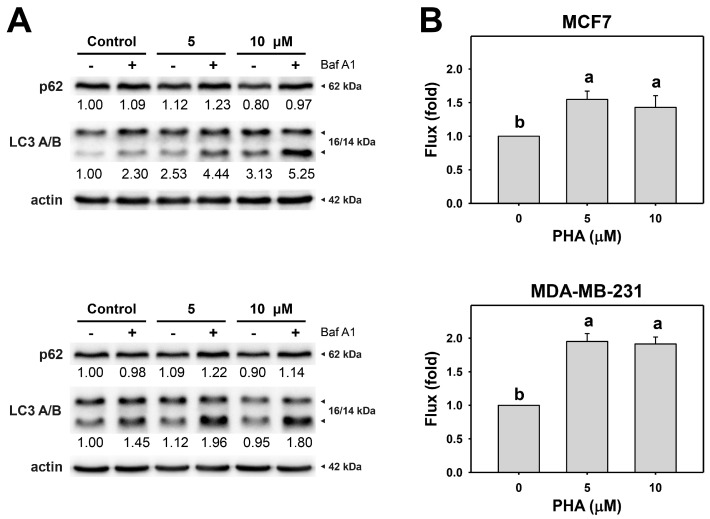
PHA induces autophagy and autophagic flux of breast cancer cells. (**A**) p62 and LC3 A/B expressions. (**B**) Autophagic flux. Cells were treated with PHA (0 (control), 5, and 10 μM) for 6 h and then co-treated with Baf A1 (control and 100 nM) for 2 h. Data, means ± SDs (*n* = 3). Data with non-overlapping lower-case letters differ significantly (*p* < 0.001 to 0.0001).

Since the autophagy inhibitor enhanced PHA-induced cytotoxicity in breast cancer cells, autophagy activity might be altered in cells. The action of autophagy was first examined using typical autophagy markers LC3B and p62 [40,41]. LC3 and p62 of breast cancer cells were assessed by immunoblot assays (Figure 2A). PHA treatment caused the accumulation of LC3B and decreased p62 in MCF7 and MDA-MB-231, indicating that PHA triggers autophagy progression in breast cancer cells.

Moreover, to precisely measure autophagic flux, the autophagy inhibitor Baf A1 was applied to assess autophagic flux in PHA-treated breast cancer cells (Figure 2B). The autophagy inhibitor Baf A1 alleviated LC3B accumulation in PHA-treated breast cancer cells. Accordingly, PHA functions as an autophagy inducer.

### 3.3. PHA Promotes Autophagy Expressions

**Figure 3:**
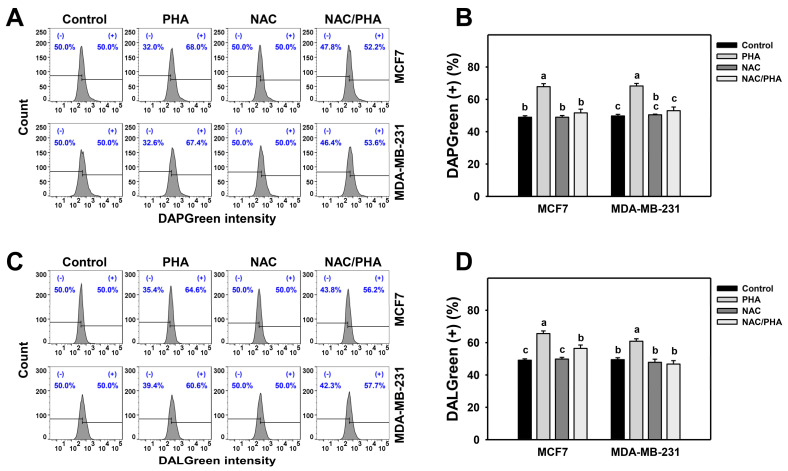
Inhibition of ROS decreases PHA-induced autophagy intensity in breast cancer cells. (**A**,**B**) Suppression of PHA-induced DAPGreen-detected autophagy by NAC. (+) indicates DAPGreen (+) (%). (**C**,**D**) Suppression of PHA-induced DALGreen-detected autophagy by NAC. (+) indicates DALGreen (+) (%) Breast cancer cells (MCF7 and MDA-MB-231) were incubated with 0.1 μM DAPGreen dye or 0.5 μM DALGreen dye at 37 °C for 30 min. Cells were pretreated with NAC (10 mM for 1 h) and post-treated with PHA (control and 5 μM for 6 h), namely NAC/PHA. Data, means ± SDs (*n* = 3). Data with non-overlapping lower-case letters differ significantly (*p* < 0.05 to 0.0001).

DAPGreen detects autophagosomes and autolysosomes, whereas DALGreen detects only autolysosomes [33,34]. PHA induced both DAPGreen (Figure 3A,B) and DALGreen (Figure 3C,D) intensities of breast cancer cells (MCF7 and MDA-MB-231), inhibited by NAC pretreatment. Accordingly, PHA functions as an autophagy inducer in an oxidative stress-dependent manner.

### 3.4. Autophagy Downregulation Promotes PHA-Induced ROS Generation

**Figure 4:**
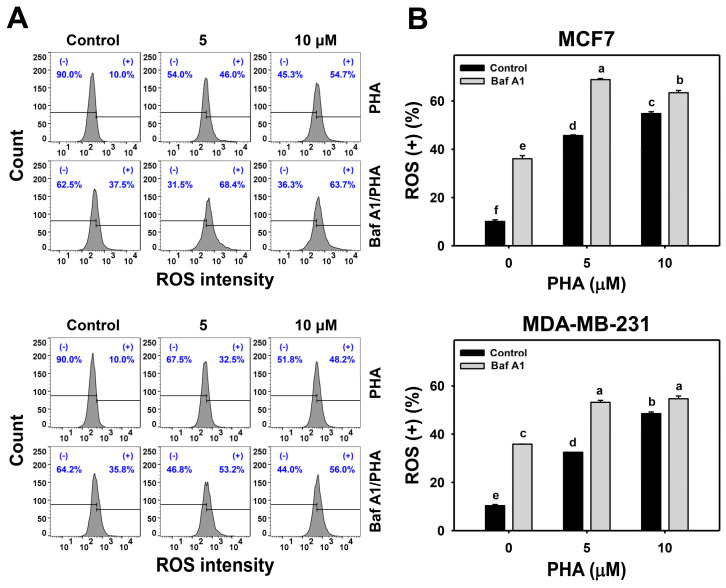

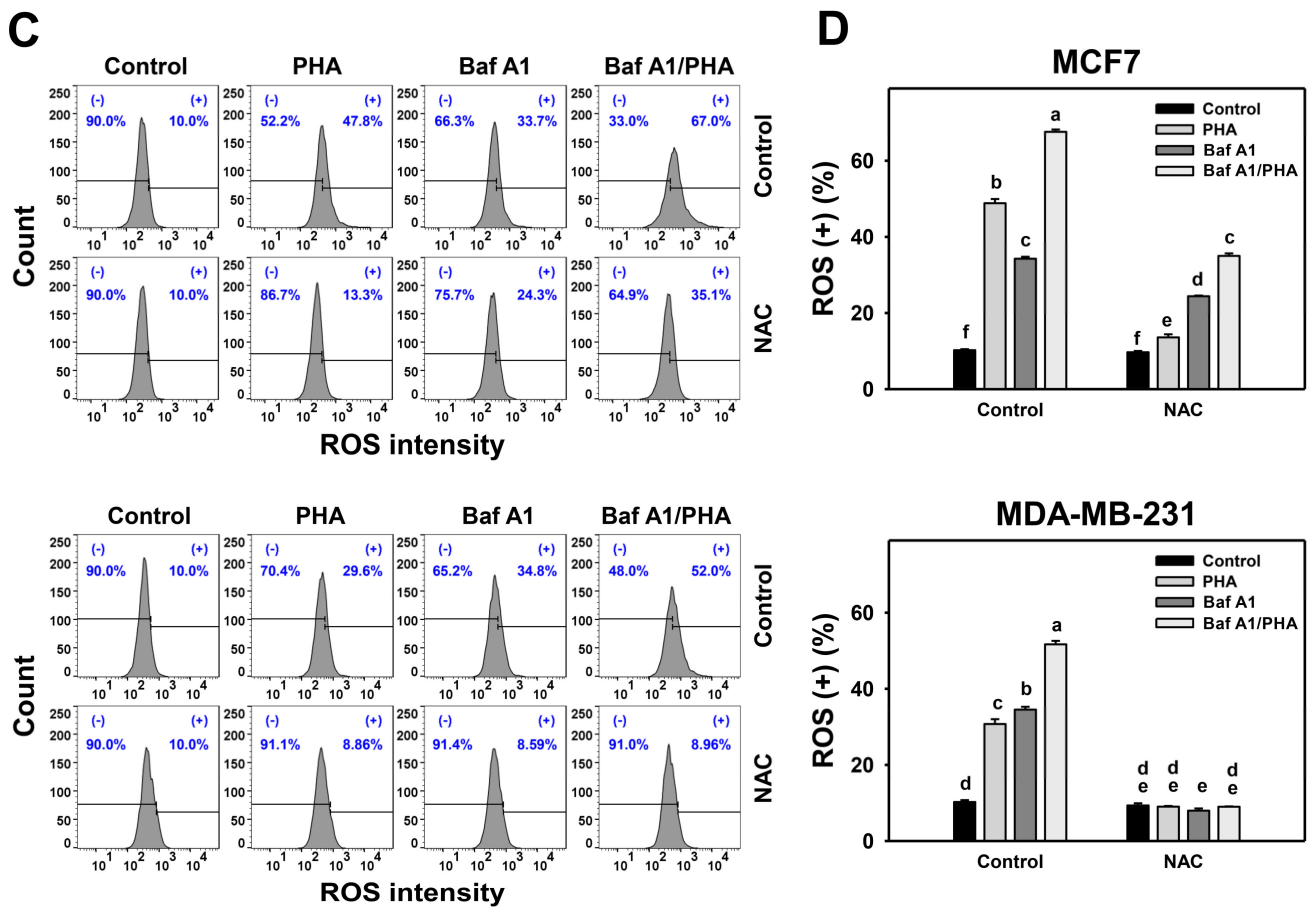
Autophagy downregulation promotes PHA-induced ROS generation in breast cancer cells. (**A**,**B**) ROS detection. Cells were co-treated with PHA (0 (control), 5, and 10 μM) and Baf A1 (0 (control) and 100 nM) for 24 h. (+) indicates for ROS (+) (%). (**C**,**D**) The impact of NAC effect on PHA-induced ROS generation. Cells were pretreated with NAC (10 mM for 1 h) and then co-treated with PHA (control and 5 μM) and Baf A1 (control and 100 nM) for a 24 h treatment, namely Baf A1/PHA. Data, means ± SDs (*n* = 3). Data with non-overlapping lower-case letters differ significantly (*p* < 0.05 to 0.0001).

ROS is induced by PHA and results in cell death. Moreover, the interplay between ROS and autophagy in various diseases has been reported previously [42,43]. To further examine the impact of autophagy on ROS production in breast cancer cells, ROS levels were detected in H_2_DCFDA staining for flow cytometry (Figure 4A,C). The ROS levels of breast cancer (MCF7 and MDA-MB-231) cells were dose-responsively increased by PHA treatment. Moreover, Baf A1 alone significantly increased ROS level. Cotreatment with Baf A1 and PHA displayed higher ROS levels than PHA or Baf A1 treatments. Accordingly, autophagy inhibition promotes the PHA-triggered ROS generation of breast cancer cells (Figure 4B).

This ROS change of PHA/Baf A1 cotreatment raises the importance of possible interaction between autophagy and oxidative stress. The ROS scavenger NAC was used to assess the impact of oxidative stress in regulating Baf A1-upregulated ROS (Figure 4C). NAC pretreatment suppressed the ROS levels of PHA and/or Baf A1 treatments of MCF7 and MDA-MB-231 cells (Figure 4D).

### 3.5. Autophagy Downregulation Promotes PHA-Stimulated Antioxidant Expressions

**Figure 5:**
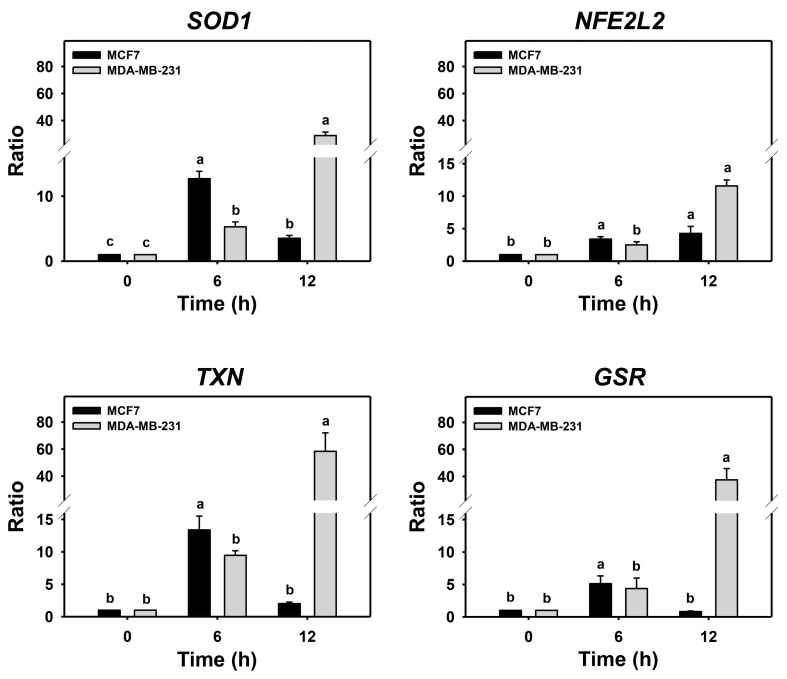
Autophagy downregulation promotes PHA-stimulate antioxidant mRNA expressions in breast cancer cells. The ratio of mRNA expressions for Baf A1/PHA cotreatment/PHA treatment was calculated to determine the impact of Baf A1 on mRNA expressions of antioxidant genes (*SOD1*, *NFE2L2*, *TXN*, and *GSR*). When the Baf A1/PHA cotreatment/PHA treatment ratio is larger than 1, it indicates that Baf A1 can stimulate mRNA expressions of test antioxidant genes. Breast cancer (MCF7 and MDA-MB-231) cells were co-treated with PHA (0 (control) and 5 μM) and Baf A1 (100 nM) for 0, 6, and 12 h. Data, means ± SDs (*n* = 3). Data with non-overlapping lower-case letters differ significantly (*p* < 0.05 to 0.0001).

Cells may turn on the defense antioxidant signaling in response to oxidative stress. Since either PHA treatment or Baf A1/PHA cotreatments induced ROS, the effects of autophagy in antioxidant gene expressions such as *SOD1*, *NFE2L2*, *TXN*, and *GSR* in breast cancer cells were assessed by real-time PCR (Figure 5). For MDA-MB-231 cells, the (Baf A1/PHA)/PHA ratio of mRNA expressions for *SOD1*, *NFE2L2*, *TXN*, and *GSR* genes was time-dependently increased. For MCF7 cells, the (Baf A1/PHA)/PHA ratio of mRNA expressions for the *NFE2L2* gene was time-dependently increased, while the mRNA expressions of *SOD1*, *TXN*, and *GSR* genes were upregulated at 6 h and declined at 12 h.

### 3.6. Autophagy Downregulation Promotes PHA-Triggered Apoptosis

**Figure 6:**
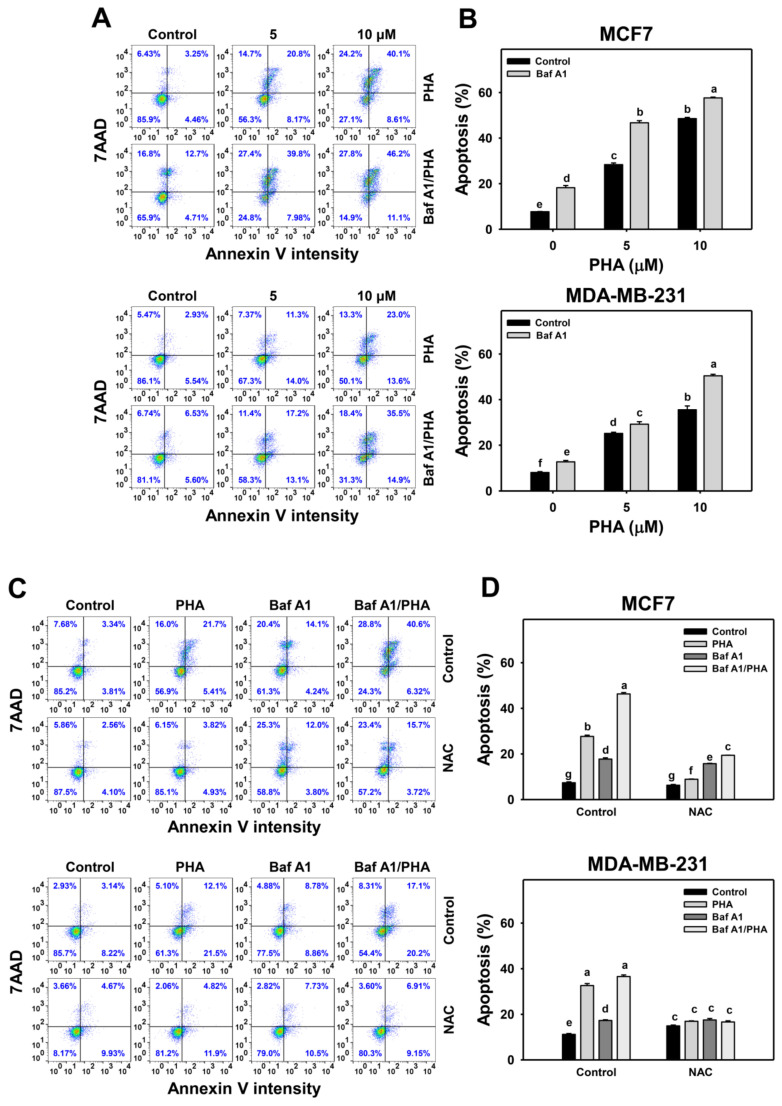
Autophagy downregulation increases the PHA-induced annexin V level in breast cancer cells. (**A**,**B**) Annexin V/7AAD detection. Cells were co-treated with PHA (0 (control), 5, and 10 μM) and Baf A1 (0 (control) and 100 nM) for 24 h. Annexin V (+)/7AAD (+/−) events indicate apoptosis (+) (%). (**C**,**D**) The impact of NAC effect on PHA-induced apoptosis. Cells were pretreated with NAC (10 mM for 1 h) and then co-treated with PHA (control and 5 μM) and Baf A1 (control and 100 nM) for a 24 h treatment, namely Baf A1/PHA. Data, means ± SDs (*n* = 3). Data with non-overlapping lower-case letters differ significantly (*p* < 0.05 to 0.0001).

In response to oxidative stress, cells may turn on autophagy and apoptosis [44], which may interact with each other [45]. To address this concern, Baf A1 was applied to assess the impact of autophagy on PHA-triggered apoptosis. An apoptosis level was detected in annexin V staining for flow cytometry (Figure 6A,C). The annexin V level of breast cancer (MCF7 and MDA-MB-231) cells was dose-responsively increased by PHA treatment. Moreover, Baf A1 alone increased the annexin V level. Cotreatment with Baf A1 and PHA displayed a higher annexin V level than PHA or Baf A1 treatments. Accordingly, autophagy inhibition promotes the PHA-triggered annexin V-detected apoptosis of breast cancer cells (Figure 6B).

This annexin V change of PHA/Baf A1 cotreatment raises the importance of possible interaction between autophagy and apoptosis in breast cancer cells under oxidative stress. The antioxidant NAC was used to assess the impact of oxidative stress in regulating Baf A1-upregulated annexin V (Figure 6C). NAC pretreatment suppressed the annexin V-detected apoptosis of PHA and/or Baf A1 treatments of MCF7 and MDA-MB-231 cells (Figure 6D).

### 3.7. Autophagy Downregulation Promotes PHA-Triggered Apoptosis Signaling

**Figure 7:**
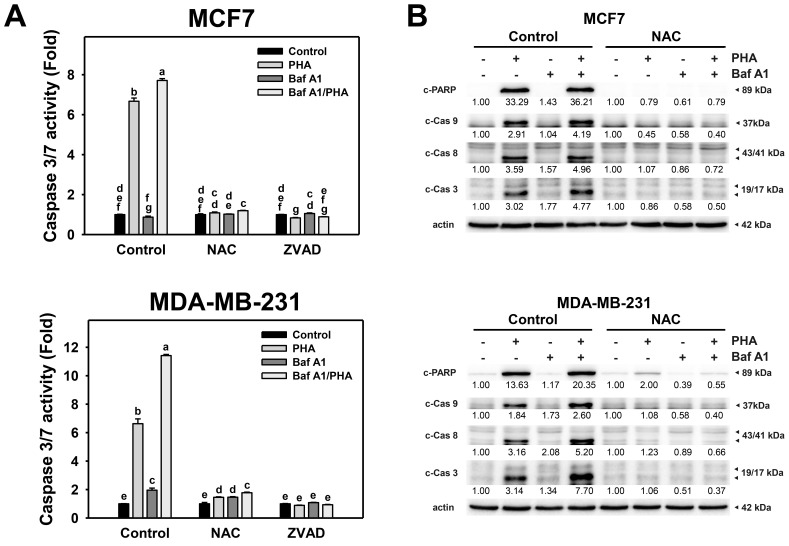
Autophagy downregulation promotes PHA-triggered activations of Caspase 3/7 and apoptotic signaling in breast cancer cells. (**A**) Caspase 3/7 activation. Cells were pretreated with NAC (10 mM for 1 h) or ZVAD (100 μM for 2 h) and then co-treated with PHA (control and 5 μM) and Baf A1 (control and 100 nM) for 24 h. (**B**) The impact of NAC effect on PHA-triggered apoptosis signaling. Cells were pretreated with NAC (10 mM for 1 h) and then co-treated with PHA (control and 5 μM) and Baf A1 (control and 100 nM) for a 24 h treatment. Data, means ± SDs (*n* = 3). Data with non-overlapping lower-case letters differ significantly (*p* < 0.05 to 0.0001).

To further validate the interplay of autophagy and apoptosis in response to PHA-induced ROS production, Caspase 3/7 activity and protein levels of active caspases were detected. The Caspase 3/7 activity of breast cancer (MCF7 and MDA-MB-231) cells was increased by PHA treatment (Figure 7A). The combination of PHA with Baf A1 induced increased Caspase 3/7 activation than a single PHA or Baf A1 treatment. Accordingly, autophagy inhibition promotes the PHA-triggered caspase 3/7 activation for apoptosis signaling of breast cancer cells. Moreover, the PHA and/or Baf A1 treatment-induced Caspase 3/7 activation was suppressed by NAC and ZVAD pretreatments.

Additionally, cleaved forms of active caspase for apoptosis signaling were used to determine the impact of oxidative stress and autophagy. This modulated the PHA-triggered apoptosis, including extrinsic and intrinsic initiators caspase 8 and 9 and executioner caspase 3 (Figure 7B). Consistently, PHA increased the cleaved forms of caspase 3/8/9 and PARP in MCF7 and MDA-MB-231 cells, while BafA1 enhanced cleaved caspase 3/8/9 and PARP. In contrast, NAC pretreatment suppressed the activation of caspase 3/8/9 and cleavage of PARP in PHA and/or BafA1 treated MCF7 and MDA-MB-231 cells, implying PHA-triggered ROS induces cytoprotective autophagy and detrimental extrinsic and intrinsic apoptosis in breast cancer cells. 

## 4. Discussion

*P. peruviana*-derived PHA was reported as a selective inhibitor in cancer proliferation and survival, likely through ROS production [12]. Autophagy is a self-eating pathway to get rid of damaged components, which maintains homeostasis in cells. Besides the cytoprotective role of autophagy in cells, recent studies also show that autophagy could be detrimental in cells in response to stresses, particularly in oxidative stress [42,43]. However, the impact of PHA on autophagy in breast cancer cells has never been investigated. Our present study reported that PHA-induced ROS production might be required for induction of cytoprotective autophagy in breast cancer.

### 4.1. PHA Tiggers ROS Generation and Autophagy Induction in Breast Cancer Cells

ROS is an initiator to trigger several pathways for survival and death in cells during various stresses [43,46]. Regarding the potential mechanisms for ROS in autophagy induction in cells when exposed to PHA, the transcriptional and post-translational regulations for ROS in autophagy induction have been reported previously. Transcription factors, such as hypoxia-inducible factor-1*α* (HIF-1*α*) and NRF2, are activated in a cell response to ROS production. HIF-1*α* and NRF2 induce autophagy-related genes, including *BECN1*, *LC3*, *SQSTM1*, *BNIP3*, and *NIX* [42]. Besides ATG genes, HIF-1α and NRF2 also turn on gene expression of antioxidant cytoplasmic proteins sestrins (SESNs) [47,48]. SESNs recruit AMPK to inactivate MTORC1 for autophagy imitation [49], while SESNs associate with ULK1 to activate SQSTM1 for autophagy processing [50]. Our current results showed that PHA induced ROS to promote cytoprotective autophagy (Figure 1, Figure 2 and Figure 3). The mechanisms of ROS involvement in autophagy activation might need further studies for elucidation.

Autophagy serves as an antioxidant pathway in cells under the high level of ROS, mainly through clearance of impaired organelles and the activation of antioxidant transcription factor nuclear factor erythroid 2-related factor 2 (NRF2) [42]. The inhibition of autophagy may enhance ROS production in cells. For example, autophagy inhibitor 3-methyladenine (3-MA) promotes high glucose-induced ROS production in adipose-derived stem cells (ADSCs) [51], while chloroquine, another autophagy inhibitor, upregulates mitochondrial ROS to enhance cisplatin sensitivity in cholangiocarcinoma cells [52]. Vacuolar-type (H^+^)-ATPase inhibitors Baf A1 and concanamycin A block the autophagosome and lysosome fusion step to increase the production of ROS in RAW 264 cells [53]. In line with previous results, our present results indicate that the inhibition of autophagy elevated ROS production and enhanced PHA-induce ROS levels in breast cancer cells (Figure 4). In contrast, ROS scavenger NAC eliminated ROS levels and autophagic activity. These results suggest that PHA-triggered ROS is required for autophagy induction, which might be a feedback loop to diminish ROS levels in breast cancer cells.

### 4.2. Interplay of Autophagy and Apoptosis in PHA-Treated Breast Cancer Cells

The interplay of ROS-mediated autophagy and death in cancer cells has been reported previously [43]. A small amount of ROS may activate autophagy signaling and allow cancer cells to survive under stressed microenvironments, such as hypoxia and starvation. Too much ROS may cause cancer cell death, particularly in treatments with anticancer drugs. Genetic and pharmacological ablation of autophagy increases overloaded ROS production and results in cancer cell death. Silencing ATG5 or treatment with an autophagy inhibitor enhances Honokiol-induced apoptosis in prostate cancer cells [54]. Inhibitors or silencing ATG7 increases Quercetin-induced ROS and leads to apoptosis in glioblastoma cells [55]. These results suggest that ROS plays a central role in regulating both autophagy and cell death. Indeed, our results showed that PHA induced autophagy (Figure 2 and Figure 3) and apoptosis (Figure 6 and Figure 7) in breast cancer cells. Autophagy inhibitor Baf A1 enhanced PHA-induced ROS levels and apoptosis. Furthermore, ROS scavenger NAC largely reduced PHA and/or Baf A1 induced apoptosis. These results indicated that PHA elevated ROS production to activate apoptosis, whereas the elevated ROS also triggered autophagy as a survival pathway, suggesting that a combinational treatment of PHA with autophagy inhibitor would be more effective for killing breast cancer cells.

In addition, ROS may induce several types of cancer cell death except for apoptosis, such as necroptosis and ferroptosis. Shikonin, a natural product isolated from *Lithospermum erythrorhizon*, induced ROS production to promote necroptosis, whereas mitochondrial superoxide antioxidant MnTBAP inhibits the expression of RIP1 and RIP3 and blocks necroptosis [56]. Moreover, ROS, in particular superoxide (O_2_^−^), is required for sodium selenite-induced ferroptosis in various cancer types, including breast cancer MCF7, glioma U87MG, and prostate cancer PC3 cells [57], while ferrostatin-1 and deferoxamine, potent ferroptosis inhibitors, block sodium selenite-induced ferroptosis. Together with previous findings, different types of cancer death might be involved in PHA-suppressed effects on breast cancer cells, which will require further studies for validation.

## 5. Conclusions

**Figure 8:**
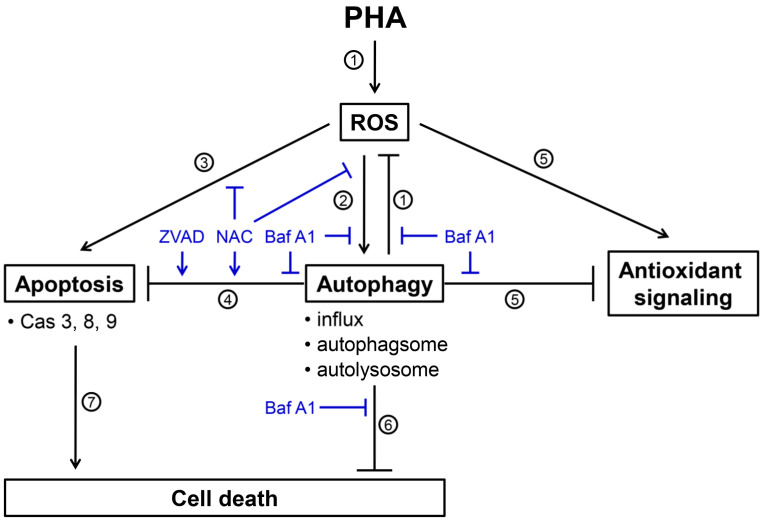
Scheme for illustrating the PHA-induced ROS, cytoprotective autophagy, and apoptosis of breast cancer cells. Several events were mentioned as follows. (**1**) PHA induces ROS generations and Baf A1 (autophagy inhibitor) inhibits autophagy-suppressed ROS to increase ROS levels (Figure 4), suggesting that autophagy exerts antioxidant effects. (**2**) PHA-induced ROS generation stimulates autophagy by increasing autophagy influx, autophagosome, and autolysosome (Figure 2 and Figure 3). These PHA-stimulated autophagy changes were suppressed by NAC (ROS inhibitor) and Baf A1 (Figure 3 and Figure 2), supporting the involvement of ROS and autophagy in this regulation. (**3**) PHA causes apoptosis associated with annexin V increment and caspases 3, 8, and 9 activations (Figure 6 and Figure 7). NAC suppresses PHA-induced apoptosis, demonstrating this apoptosis induction is ROS-dependent. (**4**) This PHA-stimulated autophagy suppresses apoptosis, as supported by the evidence that Baf A1 induces apoptosis (Figure 6 and Figure 7). This apoptosis-suppressing effect of PHA-stimulated autophagy is also activated by NAC and ZVAD (apoptosis inhibitor), supporting the involvement of ROS in this regulation. (**5**) PHA-stimulated autophagy suppresses antioxidant signaling, supported by the finding that antioxidant signaling is upregulated by Baf A1 (Figure 5). This upregulation of antioxidant signaling may be a stress response to ROS. (**6**) PHA-stimulated autophagy is cytoprotective because Baf A1 enhances the cytotoxicity of PHA in breast cancer cells (Figure 1). (**7**) Finally, PHA-induced apoptosis contributes to cell death of breast cancer cells [12]. Taken together, PHA induces ROS to promote cytoprotective autophagy, which limits its-mediated apoptosis of breast cancer cells.

*P. peruviana*-derived PHA is a potent anticancer compound for various types of cancer cells. Autophagy could be a survival or death pathway in cancer cells when exposed to anticancer drugs. The role of autophagy in PHA-induced stress remains unclear. Our current study shows that PHA-induced ROS triggers a signal required to activate both autophagy and apoptosis pathways in breast cancer cells. The inhibition of autophagy increased PHA-induced ROS levels and apoptosis. ROS scavenger NAC diminished both autophagy and apoptosis. A combinational treatment with PHA and autophagy inhibitors caused overloaded ROS to enhance the cytotoxic effects of PHA in breast cancer cells, suggesting that autophagy might serve as an antioxidant pathway and limit the killing effects of PHA in breast cancer cells. The scheme for illustrating the PHA-induced ROS, cytoprotective autophagy, and apoptosis of breast cancer cells is shown in Figure 8. Our study suggests a mechanistic insight into the cytoprotective role of autophagy in PHA-treated breast cancer cells.

## Figures and Tables

**Table 1:** Basic information for antioxidant-associated genes.

Genes	Forward Primers (5’→3’)	Reverse Primers (5’→3’)	Accession Numbers
*SOD1*	AGGGCATCATCAATTTCGAGC	CCCAAGTCTCCAACATGCCTC	NM_000454.4
*NFE2L2*	GATCTGCCAACTACTCCCAGGTT	CTGTAACTCAGGAATGGATAATAGCTCC	NM_006164.5
*TXN*	GAAGCAGATCGAGAGCAAGACTG	GCTCCAGAAAATTCACCCACCT	NM_003329.4
*GSR*	GTTCTCCCAGGTCAAGGAGGTTAA	CCAGCAGCTATTGCAACTGGAGT	NM_000637.5
*GAPDH*	CCTCAACTACATGGTTTACATGTTCC	CAAATGAGCCCCAGCCTTCT	NM_002046.7

## Data Availability

Data are contained within the article.

## Supplementary Materials

The following supporting information can be downloaded at: https://www.mdpi.com/article/10.3390/antiox11071352/s1. Figure S1: The ^1^H-NMR spectrum of commercially available PHA.
