# Supplementary material for: Physapruin A Induces Reactive Oxygen Species to Trigger Cytoprotective Autophagy of Breast Cancer Cells
Source: Antioxidants (Basel). 2022 Jul 11;11(7):1352. doi: 10.3390/antiox11071352 (PMC9311569; doi:10.3390/antiox11071352)
Supplement: Supplementary file 1 [file antioxidants-11-01352-s001.zip › antioxidants-1792707-supplementary.pdf]

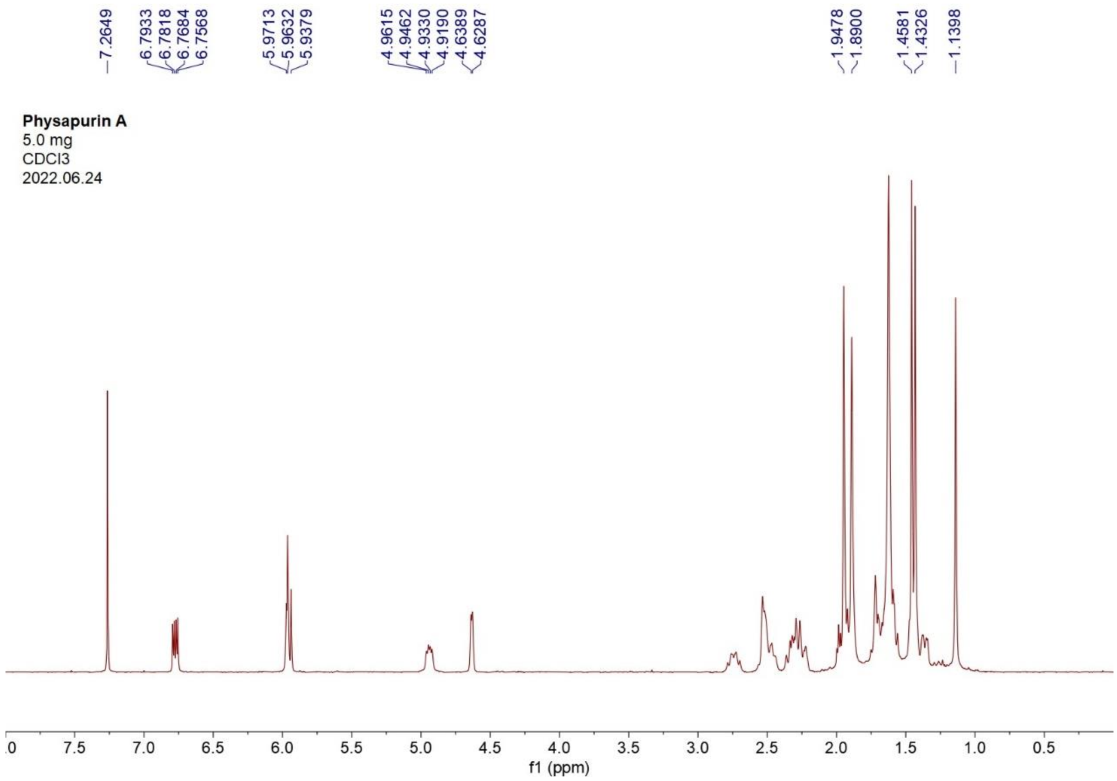

Figure S1. The ^{1}H NMR spectrum of PHA purchased from BioBioPha Co. (Yunnan, China). This spectrum matched the data for PHA in our previous work [12] and showed high purity (no impurity peaks).
